# Neural markers of social and monetary rewards in children with Attention-Deficit/Hyperactivity Disorder and Autism Spectrum Disorder

**DOI:** 10.1038/srep30588

**Published:** 2016-07-28

**Authors:** Maria Luz Gonzalez-Gadea, Mariano Sigman, Alexia Rattazzi, Claudio Lavin, Alvaro Rivera-Rei, Julian Marino, Facundo Manes, Agustin Ibanez

**Affiliations:** 1 Instituto de Neurociencia Cognitiva y Traslacional (INCYT), Laboratorio de Psicología Experimental y Neurociencias (LPEN), Fundación INECO, Universidad Favaloro, Consejo Nacional de Investigaciones Científicas y Técnicas (CONICET), Buenos Aires, Argentina; 2 Universidad Di Tella. Buenos Aires, Argentina; 3 Programa Argentino para Niños, Adolescentes y Adultos con Condiciones del Espectro Autista (PANAACEA), Buenos Aires, Argentina; 4 Centre for the Study of Argumentation and Reasoning, Faculty of Psychology, Universidad Diego Portales, Santiago, Chile; 5 Laboratorio de Neurociencia Cognitiva y Social (LaNCyS), Facultad de Psicología, Universidad Diego Portales, Santiago, Chile; 6 Laboratorio de Neuroimágenes, Universidad Nacional de Córdoba, Argentina; 7 Grupo de Neurociencia Cognitiva, Universidad de Granada, España; 8 Centre of Excellence in Cognition and its Disorders, Australian Research Council (ACR), New South Wales, Australia; 9 Center for Social and Cognitive Neuroscience (CSCN), School of Psychology, Universidad Adolfo Ibanez, Santiago de Chile, Chile; 10 Universidad Autonoma del Caribe, Barranquilla, Colombia

## Abstract

Recent theories of decision making propose a shared value-related brain mechanism for encoding monetary and social rewards. We tested this model in children with Attention-Deficit/Hyperactivity Disorder (ADHD), children with Autism Spectrum Disorder (ASD) and control children. We monitored participants’ brain dynamics using high density-electroencephalography while they played a monetary and social reward tasks. Control children exhibited a feedback Error-Related Negativity (fERN) modulation and Anterior Cingulate Cortex (ACC) source activation during both tasks. Remarkably, although cooperation resulted in greater losses for the participants, the betrayal options generated greater fERN responses. ADHD subjects exhibited an absence of fERN modulation and reduced ACC activation during both tasks. ASD subjects exhibited normal fERN modulation during monetary choices and inverted fERN/ACC responses in social options than did controls. These results suggest that in neurotypicals, monetary losses and observed disloyal social decisions induced similar activity in the brain value system. In ADHD children, difficulties in reward processing affected early brain signatures of monetary and social decisions. Conversely, ASD children showed intact neural markers of value-related monetary mechanisms, but no brain modulation by prosociality in the social task. These results offer insight into the typical and atypical developments of neural correlates of monetary and social reward processing.

When people act prosocially (e.g., cooperating with others), they experience positive reward and engage reward circuits that overlap with those that are engaged upon the receipt and consumption of monetary wins^1^. Similarly, disloyal social behavior (e.g., betrayal) is experienced as a negative outcome, and such experiences are similar to those expressed after monetary losses. Specifically, value-related brain processes that are typically activated during monetary decisions (i.e., the selection of options that imply wins and losses)^2^ are also modulated during social behavior (e.g., charitable donations, social cooperation, and prosociality)^3^,^4^,^5^,^6^. Thus, different choice outcomes, such as wins and losses (Monetary Decision Making, MDM) and cooperation and betrayal (Social Decision Making, SDM), would involve similar neural modulations by rewards and punishments. However, no previous study has contrasted these two pathways in terms of the same neural marker of reward processing. The current study aimed 1) to identify a neural marker that was capable of indexing monetary and social rewards; and 2) to use this proxy to examine whether these brain processes are affected in children with Attention-Deficit/Hyperactivity Disorder (ADHD) and Autism Spectrum Disorder (ASD). Toward the first aim, we focused on the feedback Error-Related Negativity (fERN, also called mediofrontal-negativity-MFN, or feedback related negativity-FRN) as an electrophysiological marker of value-related brain processes that underlie both MDM and SDM. This component is primarily generated by the Anterior Cingulate Cortex (ACC)^7^, which is a crucial region for the processing monetary rewards^8^ and the motivational value of social interactions^3^,^5^,^9^,^10^. The fERN has exhibited a greater negative deflection following losses compared with wins during MDM tasks^8^,^11^,^12^. Additionally, SDM tasks that have been used to investigate fairness evaluation^13^,^14^ and social rejection^15^ have provided evidence of greater fERN responses during undesirable social interactions. Based on this evidence, we hypothesized that the fERN response and its associated ACC source activation would be modulated in the same manner by monetary losses during an MDM task and non-cooperative choices (i.e., betrayal decisions) during a SDM task, even though the latter choices do not imply financial losses.

To address the second aim of this study, we investigated how the fERN, which served as a neural marker of SDM and MDM, was modulated in two neuropsychiatric disorders with apparently different profiles of deficits in these processes. ADHD children are characterized by impaired neural reward processing during MDM, presenting abnormal brain activation to rewards and penalties^16^,^17^, but no previous study has reported on the neural correlates of SDM in this population. However, because basic motivational/reward processing is affected in ADHD^16^,^17^,^18^, we hypothesized that the value-related brain markers of both MDM and SDM would be compromised in these children. Conversely, only abnormal correlates of SDM have been reported in ASD individuals^19^,^20^ who exhibit a preference for self-centered rather than prosocial choices^21^,^22^. In contrast, intact MDM reward processing has been observed in these children^23^,^24^,^25^. Accordingly, we hypothesized that the ASD children would exhibit intact feedback-related MDM modulation but that their neural markers of atypical SDM would be modulated by the participant’s own benefit rather than prosociality.

We tested these hypotheses using high-density electroencephalography (hdEEG) while ADHD, ASD, and control participants performed a MDM task and observed a SDM paradigm. For the MDM, we employed a children’s version of the Iowa Gambling Task (IGT)^26^ in which the participants win and lose money by selecting cards from high- and low-risk options. For the SDM, we used a modified version of the Prisoner’s Dilemma Game (PDG)^27^. The participants observed a game between two players who either cooperated or betrayed with each other to gain points. We specifically configured the game such that betrayal options resulted in greater monetary payoffs than did cooperation options.

In summary, our hypotheses were the following: (1) the typically developing children would exhibit greater fERN responses (and associated ACC source activation) in response to losses compared with wins in the MDM task and in response to betrayal compared with cooperation options in the SDM task; (2) due to a general deficit in reward mechanisms, the ADHD children would exhibit reduced fERN modulation and related ACC source activation in both MDM and SDM tasks; and (3) in the ASD children, we expected to observe normal fERN modulation and associated ACC source activation in the MDM task and fERN/ACC patterns that were opposite to those of the controls in the SDM task, i.e., the responses would be driven by monetary rewards rather than prosocial motivation.

## Materials and Methods

### Participants

Sixty-seven participants, including 22 typically developing participants (14 boys and 8 girls), 19 children diagnosed with ADHD (13 boys and six girls), and 28 with ASD (27 boys and 1 girl), were recruited. Some participants were excluded from the data analysis (see details in Table 1 and section 1 of the Supplementary Information). The individuals in the ADHD and ASD groups were selected from 60 outpatients of the Institute of Cognitive Neurology (INECO) and related institutions based on the following inclusion criteria: (1) age between 8 and 15, similar to previous studies^28^,^29^ and (2) an ADHD or ASD diagnosis according to the Diagnostic and Statistical Manual of Mental Disorders, fifth edition (DSM-5)^30^. These children were evaluated during interviews for admission to the specialized clinic of developmental disorders, during which they underwent a detailed examination that included neuropsychiatric, neurological, and neuropsychological evaluations. To quantify the ADHD symptom presentations, we used the Conners’ Parent Rating Scale Revised: Short form (CPRS-R:S)^31^ (see Table 1). To measure the ASD symptoms, we used the Developmental, Diagnostic and Dimensional Interview (3Di)^32^. The 3Di is a widely used standardized diagnostic instrument that was designed according to the current conceptualization of ASD as a dimensional disorder^33^. Note that some ASD children presented high ADHD symptoms (see Table 1). However, the co-occurrence of symptoms is consistent with the current diagnosis criteria for ASD^30^. Moreover, this overlap did not affect the distinctive pattern of results observed in each patient group (see details in section 2 of Supplementary Information). The ADHD and ASD subjects took no medications during the 48 hours prior to the hdEEG recordings.

Twenty-five control participants were recruited from neighboring schools. The exclusion criteria for this group were the following: (1) age outside the range of 8 to 15 and (2) a history of intellectual disability or neurological or psychiatric diseases. Using group-wise matching criteria, 22 of these participants were selected to form a control group that was matched for age and fluid intelligence (Raven’s Progressive Matrices Test, RPMT^34^) to both the ADHD and ASD groups (see Table 1). Only the RPMT was used as an intelligence matching variable due to time constraints and because it is a more reliable measure of intelligence in the ASD population than complex verbal intelligence scales^35^,^36^. All participants provided a verbal informed assent, and a parent, next of kin, caretakers, or guardian gave written informed consent on behalf of the child enrolled in this study. All protocols were performed in accordance with relevant guidelines and regulations of the Declaration of Helsinki. The study was approved by the ethics committee of INECO.

### MDM: IGT for children (IGT-C)

**Figure 1:**
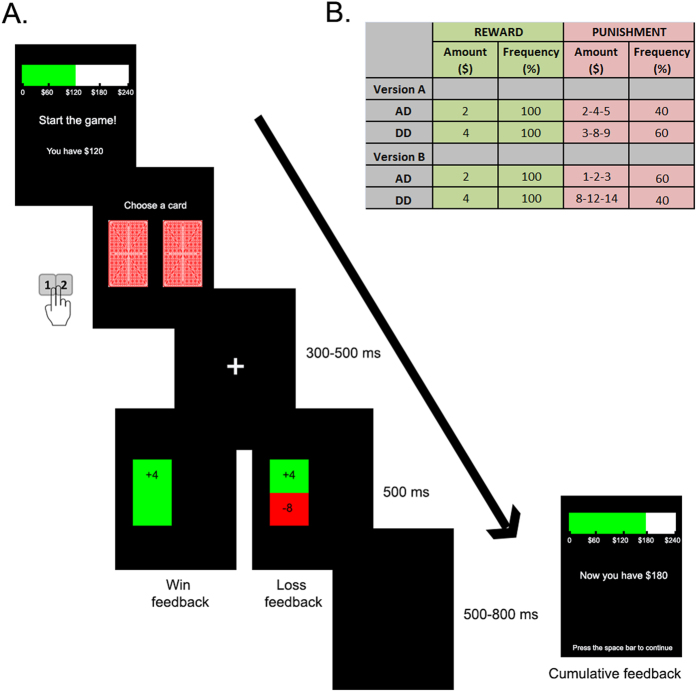
IGT-C. (**A)** IGT-C trial sequence: Each version started with the presentation of the initial capital ($120). Each trial began with a screen showing two decks that was displayed until the participant responded. After the response, a fixation cross window was replaced with the outcome (feedback onset for the fERN). Finally, a black screen appeared and indicated the start of the next trial. After 20 choices, an outcome display revealed the cumulative feedback (see details in section 3 of the Supplementary Information). **(B)** Table illustrating the distributions of win and loss across the decks and versions of the IGT-C (AD: advantageous deck, DD: disadvantageous deck).

We adapted the computerized four-deck IGT to make it suitable for children and for the ERP analysis (IGT-C). The task included two versions with two decks each. The IGT-C has been validated previously in typically developing children through behavioral and psychophysiological measures^26^. Figure 1A illustrates an example of a trial sequence. The participants were instructed to select a card from either the left or the right deck to maximize their initial capital ($120). Each time that a card was selected, a feedback display revealed the magnitude of win or loss. After 20 choices, cumulative feedback was presented. The task was completed after the 8^th^ presentation of cumulative feedback. All participants performed both versions (160 trials each) and were blind to the distribution of wins and losses across decks and versions (see details in section 3 of the Supplementary Information).

Each version of the IGT-C task included two decks that differed in terms of long-term profit (advantageous and disadvantageous) and loss frequency (high and low). Both versions contained an advantageous deck (AD) and a disadvantageous deck (DD). The amount and frequency of wins were constant across the versions, while the amounts and frequencies of losses differed across decks and versions (see Fig. 1B). The number of cards selected in each deck and version were taken as behavioral measures to compare differences between groups regarding task understanding and attention. Given that children’s main strategy in the IGT is to avoid both the DD and options with high-loss frequency^26^,^37^, we contrasted the fERN responses between wins and losses following the selection of these options.

### SDM: PDG for children (PDG-C)

**Figure 2:**
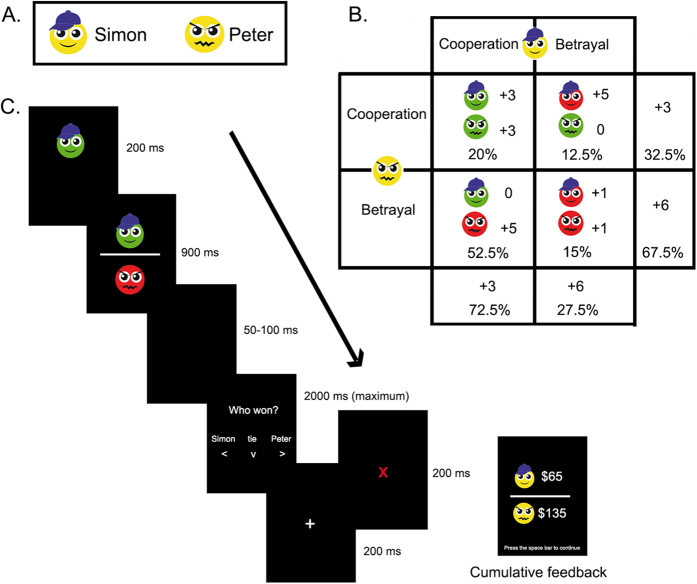
PDG-C. **(A)** PDG-C players: Simon (fair player) and Peter (unfair player). **(B)** The matrix shows the four possible outcomes resulting from the players’ interactions (see details in section 4 of the Supplementary Information). **(C)** PDG-C trial sequence: The task began with the decision of one player followed by the other player’s choice (feedback onset of the fERN). Next, a black screen was replaced with a question about what had happened in the trial. Finally, a fixation cross indicated the start of the next trial. In cases of wrong answers, a red cross appeared between the question and the fixation cross window. Every 40 trials, an outcome display revealed the cumulative feedback.

We adapted the PDG^27^ to be suitable for children and for the ERP analysis (PDG-C). In the PDG-C, the participants observed a virtual game between two players who independently chose either to cooperate with each other or to betray each other. Each player was awarded points in a manner that depended on the interaction between both players’ choices. Unknown to the participants, the game was rigged such that one of the players (Simon, the fair player) mostly cooperated (72.5%) and rarely betrayed (27.5%) and the other player (Peter, the unfair player) most frequently betrayed (67.5%) and seldom cooperated (32.5%, see Fig. 2A,B). Through the task instructions, we manipulated the participant’s identification with the fair player by indicating that they would receive a reward according to the points accumulated by this player. The fERN is also modulated when one observes/identifies with other people’s actions and observes the outcomes that would have resulted from one’s own decisions. However, the amplitude of the fERN is lower during observation than during active tasks^38^,^39^. Accordingly, we manipulated the task to measure participant’s fERN modulation over the fair player’s choices^38^. Importantly, the cooperative choices from the fair player resulted in less gain for the participant (3 points) than did betrayal decisions (6 points). However, because we expected a prosocial bias in the control participants in the PDG^3^, the betrayals would generate greater fERN signals than were the cooperative choices.

Each trial began with the decision of one player followed by the other player’s choice (see Fig. 2C). The players’ faces were shown in green when they cooperated and in red when they betrayed. After both players’ decisions, each trial included a question about the outcome (Who won?, see Fig. 2C, panel 4). Only correct responses were processed in the data analyses and participants with extreme omission errors (>70%) in their responses to control questions were excluded from these analyses, as this may reflect poor attention/understanding of task instructions. The percentage of correct responses was used to index participants’ attentiveness and task understanding.

After 40 choices, an outcome display revealed the cumulative feedback. The task was completed after the 4^th^ outcome display (160 trials). The participants were blind to the number of trials and the frequencies of the outcomes (see details in section 4 of the Supplementary Information).

### hdEEG data collection and preprocessing

During the experiment, 128-channel hdEEG signals were recorded using a Biosemi amplifier and sampled at 1024 Hz. The data were downsampled to 256 Hz, and bandpass filtered at 0.5 and 20 Hz. The Biosemi reference system was recorded via the CMS electrodes, and the signals were then algebraically re-referenced to averaged mastoids off-line. The epochs were extracted between −200 ms and 800 ms relative to the feedback onset: 1) in the IGT-C the outcome of the choice selection (after the fixation cross window); and 2) in the PDG-C, following previous studies^40^,^41^, after both players’ faces. In both tasks, we focused on the fERN, which is primarily sensitive to action-outcome contingencies. Thus, the onset of the fERN was set on the outcome of the decision in place of the stimulus onset. Furthermore, the epochs were baseline-corrected relative to the mean activity during the −200 ms to 0 ms window before the feedback onset. Data containing excessive eye movement or muscular artifacts were rejected via a quasi-automated procedure (see details in section 5 of the Supplementary Information).

### Data analysis

Behavioral measures were analyzed with a repeated measures ANOVA. Offline EEG processing and analyses were conducted using the Matlab software. Artifact-free epochs were averaged for each experimental condition to obtain the ERPs. The number of rejected trials in each task was similar among groups and conditions (Supplementary Table S1). The fERN component was quantified at the Fz electrode in the 250 and 400 ms window following the feedback onset. In the IGT-C, we compared within-group differences in this component between the win and loss conditions from the DDs and high punishment frequency options. In the PDG-C, we performed within-group contrasts of the feedback after cooperative versus betrayal choices that the fair player made. The epochs in each condition included two options (see Figs 1B and 2C), which were averaged subject-wise. Thus, each condition included two options per subject. Although we have a sufficient number of trials to measure optimal fERN^42^, the number of trials differed between some conditions. Even though these differences did not seem to affect the different pattern of results between groups, future research is needed to further investigate how these differences may impact the fERN.

The fERN differences between conditions in each group were assessed for significance via Monte Carlo permutation tests with bootstrapping^43^. This method does not require one to make assumptions about the normal distribution of the data and offers a straightforward solution for relatively small samples by performing multiple hypothesis testing^44^. Combined data from each condition was randomly partitioned by sampling with replacement from the original sample and analyzed using a *t*-test. This process was repeated 5000 times to construct the *t*-value distribution under the null hypothesis (*p* < 0.05). We plotted the temporal extents of the significant differences within the window of interest and reported the Monte Carlo *t* and *p* values at the time point of the maximal differences between conditions. Cohen’s *d* was employed as a measure of effect size for the significant effects.

To further explore the neural mechanism underlying fERN modulation, we reconstructed the cortical sources of the fERN responses using Brainstorm^45^. Following previous studies in children and adolescents^29^,^46^, we first calculated a forward model using the symmetric boundary element method from OpenMEEG^47^ on the cortical surface of a template MNI brain (colin 27) with a 1-mm resolution. This method uses three layers: scalp, inner skull, outer skull, plus the cortical surface. In the next step, we estimated source activation through an inverse model. We used a depth-weigthed linear L2-minimum norm method^48^, in which source orientation was constrained orthogonally to the cortex surface with a 0.8 weigh exponent. The grand-average activation values of the fERN, subtracted between conditions (i.e., loss-minus-win in the IGT-C and betrayal-minus-cooperation in the PDG-C) were calculated and plotted on the cortical maps. The ACC region was selected based on previous studies of fERN^49^,^50^ and then identified visually in the cortical map of the control group. Supplementary Table 2S shows the coordinates of the maximum peak of activation of this ROI in each group. Afterwards, the ROI (covered by 40 vertices and 782 cm^2^) was multiplied in the grand-average cortical maps using the forward model (recording simulation function). Finally, subject-wise activation time courses were extracted by averaging activity within this region and then compared between groups using tests for nonstandard distributions (the Mann-Whitney test and Cohen’s *r* for effect sizes).

Lastly, to control the influence of demographic variables (age and gender) on EEG results (fERN modulations and ACC activation), we performed within-group correlations between age and EEG measures (both ERP and source space). Also, Mann-Whitney tests were used to contrast differences in gender regarding these measures. No differences between groups were observed in age and gender in the EEG results (see Supplementary Information, section 6, Tables S3 and S4).

## Results

### Behavioral measures

As expected, no significant group differences were observed in the number of cards in the IGT-C and the percentage of correct responses in the PDG-C. Thus, groups exhibited no differences in performance, attention, or task comprehension (Supplementary Information, sections 3 and 4).

### MDM (IGT-C)

**Figure 3:**
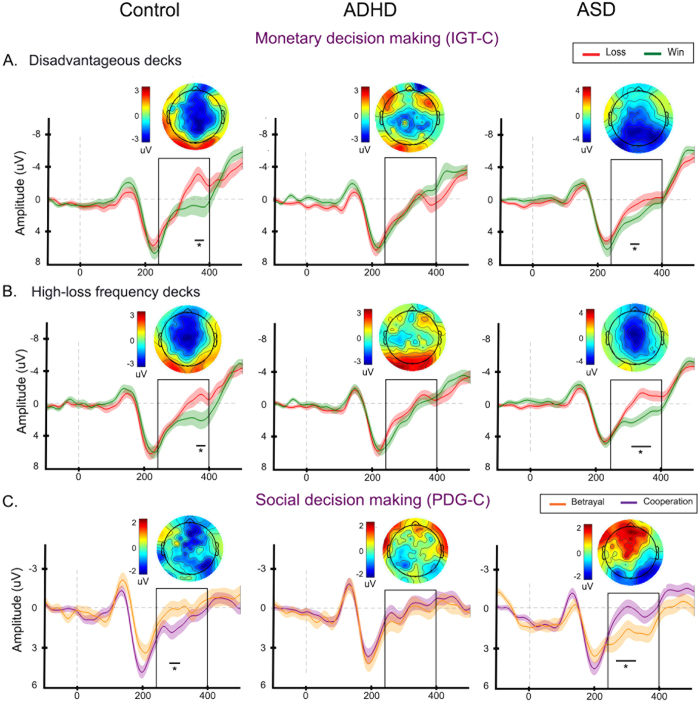
FERN modulations in the MDM and SDM tasks. ERPs of each group for: **(A)** the win and loss conditions from the disadvantageous options of the IGT-C, **(B)** win and loss conditions from the options with high-loss frequency in the IGT-C, and **(C)** the cooperative and betrayal conditions in the PDG-C. The plots at the top of each panel show the spatial topography of the subtraction between conditions (loss-minus-win in the IGT-C and betrayal-minus-cooperation in the PDG-C) at the time point of maximal difference reported in Monte Carlo permutations. The plots at the bottom show grand-average ERP time courses at the Fz electrode in microvolts. Shaded bars around the ERPs indicate s.e.m. The thick, black horizontal lines indicate the temporal extent of the significant differences between the conditions. The black rectangular box indicates the fERN time window within which the conditions were compared.

The typically developing children exhibited greater fERN responses to losses than to wins in the MDM task. This effect was observed after the selection of both types of choices in the IGT-C (DDs at a latency of 347–382 ms: *t* = −2.60, *p* = 0.01, *d* = 0.36, and high-loss frequency decks in the range of 375–386 ms: *t* = −2.42, *p* = 0.02, *d* = 0.27; see Fig. 3A,B). However, no differences between conditions were observed in the ADHD group. Regarding the ASD individuals, the fERN responses for losses were greater than those for wins following both types of choices in the IGT-C (DDs at a latency of 300 and 320 ms: *t* = −1.97; *p* = 0.048, *d* = 0.29; high-loss frequency decks in the window of 292 to 390 ms: *t* = −3.86; *p* = <0.001, *d* = 0.78; see Fig. 3A,B).

**Figure 4:**
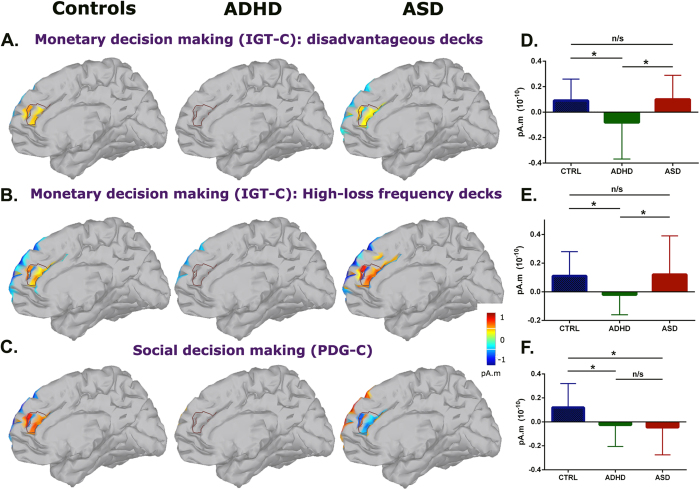
Source reconstruction for the MDM and SDM tasks. The groups’ cortical activations in the right ACC (fERN window) for: **(A)** the loss-minus-win condition for the disadvantageous options of the IGT-C**, (B)** the loss-minus win condition for the options with high-loss frequency in the IGT-C, and **(C)** the betrayal-minus-cooperation conditions from the PDG-C. The means and s.d of the ACC ROI for each group for: **(D)** the loss-minus-win condition for the disadvantageous options of the IGT-C, **(E)** the loss-minus win condition for the options with high-loss frequency in the IGT-C, and **(F)** the betrayal-minus-cooperation conditions from the PDG-C (see details in section 2.5).

The source reconstruction analysis revealed that during the 250 to 400 ms window, the controls exhibited right ACC activation following choices from the DDs and options with high-loss frequency (see details in section 3.5). However, no such activation was observed in the ADHD group. The ASD individuals exhibited ACC activation that was similar to that of the controls (see Fig. 4A,B). Thus, significant differences in these sources were observed between the control and ADHD groups (CTRL>ADHD; DDs: *U* = 76.00, *p* = 0.02, *r* = 5.83; high-loss frequency: *U* = 82.00, *p* = 0.04, *r* = 5.83) and between the ASD and ADHD participants (ASD>ADHD; DDs: *U* = 107.00, *p* = 0.01, *r* = 6.56; high-loss frequency: *U* = 121.00, *p* = 0.02, *r* = 6.56). No significant differences were observed between the control and ASD groups (see Fig. 4D,E and details in Supplementary Table S5).

Our results revealed that the ADHD children failed to exhibit neural markers of control monitoring in the ACC in response to high-risk choices (i.e., the DDs and options with high-loss frequency). These results suggest that value-related brain systems were atypical in these children. In contrast, both the controls and ASD participants exhibited intact feedback-related neural markers in response to MDM.

### SDM (PDG-C)

As expected, the fERN responses of the control children were modulated by their prosocial interest rather than their monetary profit. Accordingly, Fig. 3C shows that this group exhibited greater fERN responses to the betrayal options compared with the cooperative options at a latency of 280–305 ms (*t* = −2.24, *p* = 0.02, *d* = 0.70). The ADHD participants exhibited no significant differences between these options. Opposite to the controls, the ASD children exhibited greater fERN responses to the cooperative options compared with the betrayal options between 253 and 330 ms (*t* = −2.17, *p* = 0.03, *d* = 0.44).

Similarly, the source reconstruction analyses revealed that during a latency of 250 to 400 ms, the control participants exhibited a right ACC activation in the PDG-C (see details in section 3.5). No activation in this source was observed in the ADHD group. Remarkably, the ASD group exhibited an activation pattern that was opposite to that of the controls, i.e., a negative ACC source indicated greater activation for cooperative compared with betrayal options (see Fig. 4C). Therefore, significant differences in the ACC sources were observed between the control and ADHD groups (CTRL>ADHD: *U* = 90.00, *p* = 0.04, *r* = 5.91) and between the control and ASD participants (CTRL>ASD: *U* = 101.50, *p* = 0.01, *r* = 6.32) (see Fig. 4F and details in Supplementary Table S5).

These results revealed that during the observation of social decisions, differential neural markers of feedback processing related to the ACC were observed between the groups. First, the control participants exhibited greater brain error monitoring signals for non-social options (i.e., betrayal) that did not imply more losses for them and instead, generated more gains. The ADHD children exhibited no differential fERN modulation between these options. The absence of neural modulation during both the MDM and SDM tasks in the ADHD children is suggestive of a general reward deficit in value-related brain mechanisms. Conversely, the neural monitoring signals in the ASD children were modulated by the participants’ monetary profit rather than their social motivation (i.e., greater fERN responses and ACC activations for the cooperative options).

## Discussion

In the current study, we tested the hypothesis that both social and monetary rewards elicit motivational relevance, as indexed by a common value-related brain mechanism^1^, and that these processes would be differently affected in children with ADHD and ASD. Our results revealed that the typically developing children exhibited fERN modulations and ACC source activations for both monetary (loss>win) and social options (betray>cooperate). However, ADHD subjects exhibited reduced early cortical modulation during both monetary and social options, whereas the ASD subjects exhibited intact value-related brain markers of monetary rewards and exhibited an abnormal (inverted) modulation for social rewards. These findings suggest atypical neural processing of both monetary and social rewards, as observed in the ADHD children. However, abnormalities in brain processing of SDM could arise without deficits in the monetary value-related brain process, as observed in the ASD children.

In the present study, we contrasted the same neural markers of reward processing (i.e., the fERN and the related ACC source) in monetary and social decisions. In the typically developing children, the observed fERN modulations in the loss>win and betrayal>cooperation conditions suggested that the brain reward system responded to monetary losses and in the same manner in which this system alerted the participants of the negative values of disloyal social interactions, i.e., the observation of betrayal decisions. Furthermore, these results demonstrated that during the social options, the fERN responses and ACC-related source activities were modulated by prosocial interest rather than the individuals’ self-centered benefits. Thus, although the cooperative options meant greater losses for the participants, the betrayal options generated greater fERN responses. These results are aligned with decision making frameworks that propose that socially normative principles, such as being fair and cooperating with others, influence the basic value system when these principles are at odds with one’s own financial rewards^1^,^6^. Thus, equal distribution of rewards^6^,^51^ and cooperative behavior^3^,^52^ activate areas associated with the reward circuitry, even when the latter decisions result in financial costs to the participant. Similarly, charitable donations^5^ and decisions to lose money to punish violators of social norms^53^ activate brain regions that encode monetary rewards. In summary, these previous results in combination with our own provide evidence supporting the idea that social cooperation involves neural valuation processes that partially resemble those underlying monetary rewards.

We also tested neural correlates of both monetary and social markers of reward processing in ADHD children who are known to typically exhibit abnormalities in the reward circuitry^16^,^17^. No significant fERN differences between the win/loss and cooperation/betrayal options as well as reduced ACC task-related activations were observed in these children. Although reduced fERN modulations during monetary choices^54^,^55^ have previously been reported in ADHD, to our knowledge, this is the first study to report similar deficits in the neural correlates of social reward processing. Furthermore, these findings suggest that the abnormalities in basic brain reward processing in individuals with ADHD can be extended to the neural processing of social interactions. We propose that the absence of fERN modulations in the ADHD children may be suggestive of an atypical brain reward processing that may reflect different cognitive strategies to solve the task, rather than an impaired SDM ability in these children. Although the literature examining SDM in ADHD is minimal, one behavioral study reported difficulties in ADHD adults in adapting their behavior to the fairness of a partner^56^. Similarly, emerging evidence indicates social cognition difficulties in ADHD^57^,^58^. Therefore, our results may suggest that brain signatures of reward processing abnormalities could participate in social cognition impairments in ADHD children. Future studies should further explore this hypothesis.

In contrast to the ADHD children, the ASD children exhibited normal neural modulations during MDM. These results confirm the intact processing of monetary rewards in ASD^23^,^24^. Moreover, these children exhibited a fERN modulation and associated ACC activation for social rewards, whereas the direction of this modulation was inverted in these children compared with the controls. Thus, the fERN responses were modulated by the participants’ own financial rewards in a manner similar to that observed during the monetary choices that was independent of the social normative values of the options. These findings suggest that the brain error monitoring system in ASD children alerted the participants to losses rather than to disloyal interactions (betrayal options). Consistently, the ASD subjects prefer betrayal over cooperative decisions in the PDG^21^, make less charitable donations^22^, and are less influenced by social normative principles than control subjects during social choices. Our results are in accordance with those of previous studies^23^,^24^ that have demonstrated reduced neural underpinnings of reward anticipation for social stimuli in ASD. This abnormal pattern could be attributable to difficulties in forming reward representations for social stimuli in ASD^59^. Consequently, the neural underpinnings of social decisions may reflect different strategies to solve the task, i.e., focus on individuals’ own financial interest rather than prosocial norms. Alternatively, self-centered processing of social choices could be explained by difficulties in understanding other’s beliefs and emotions (i.e., theory of mind). Futures studies should explore these alternative explanations of social reward abnormalities in ASD.

Some limitations of this study should be acknowledged. First, our sample size was relatively small which generates the potential problem of the low statistical power of small studies (see details in ref. 60). However, we followed some of the current guidelines to deal with small samples^60^ such as selecting appropriate statistical methods^43^ and reporting all data exclusions and manipulations. In addition, our sample size was not smaller than those of previous studies^29^,^46^. Nevertheless, even taking into consideration the above points, we can not rule out that the sample size may biased the results. Further replications in larger samples should confirm the observed effects. Second, although our participants had a relatively wide age range and were unevenly distributed in terms of gender, neither variable was significantly associated with the EEG measures (Supplementary Information, section 6). Although previous behavioral studies have found gender differences in both the IGT^61^ and PDG^62^ tasks, to our knowledge, no studies to date have examined gender differences in fERN modulation or ACC activation during these tasks. Future studies should explore this issue further. Third, even though our results suggest that high ADHD symptoms in the ASD sample did not affect that group’s results, future studies should explore these processes in cases of comorbid ASD and ADHD. Fourth, we modified the original IGT and PDG to tailored ERP paradigms aimed to assess the neural correlates of MDM and SDM. Thus, similar to previous reports^25^,^54^,^63^,^64^, group differences in the ERP markers were observed in absence of behavioral indicators of these processes. This suggests that neural correlates are not explained by potential differences in participants’ attentiveness or task understanding. Group differences in neural markers could reflect subtle or subclinical impairments that may not have been evident in behavioral measures^63^,^65^. Alternatively, these differences may reflect alternative or compensatory cognitive strategies to successfully solve these tasks^66^,^67^. Future studies should explore these different interpretations by using tasks with more robust behavioral measures. Finally, although the participants were rewarded according to the fair player choices to ensure their engagement with the game, futures studies should assess the neural processing of SDM through active games that include real partners^68^.

In sum, in the present study, we drew from current models of brain signatures of reward processing in different contexts^1^. Consequently, we observed similar neural processing of monetary and social rewards in typically developing children, deficits in both processes in ADHD children, and abnormal brain processing of social cooperation with preserved monetary reward processing in ASD children. These results provide evidence that confirms that MDM and SDM induce similar activities in the brain value system. Moreover, these shared value activations rely on other domain-specific brain regions in social versus monetary contexts. These findings suggest that while typically developing children exhibit brain modulation for the motivational values of money and cooperative interactions, these processes were differentially affected by neurodevelopmental disorders. Thus, these results could offer insights into the brain mechanisms underlying the typical and atypical development of monetary and social reward processing and could open promising avenues for exploring these neural atypicalities in many other neuropsychiatric conditions.

## Additional Information

**How to cite this article**: Gonzalez-Gadea, M. L. *et al*. Neural markers of social and monetary rewards in children with Attention-Deficit/Hyperactivity Disorder and Autism Spectrum Disorder. *Sci. Rep.* **6**, 30588; doi: 10.1038/srep30588 (2016).

## Supplementary Material

Supplementary Information

## Figures and Tables

**Table 1:** Means (s.d) and group differences in demographics and diagnosis symptoms.

	Monetary decision making (IGT-C)				Social decision making (PDG-C)			
	Control	ADHD	ASD	p*	Control	ADHD	ASD	p*
N sample	21	18	28	—	19	17	22	
Gender (males:females)	13:8	12:6	27:1	—	11:8	12:5	21:1	
Matching measures								
Age	11.43 (2.40)	11.67 (2.45)	10.39 (2.06)	0.13	11.19 (2.33)	11.42 (2.52)	10.50 (1.99)	0.17
Fluid intelligence	39.10 (8.53)	39.89 (8.18)	40.14 (9.39)	0.92	38.32 (8.42)	39.88 (8.70)	39.86 (10.23)	0.84
ASD symptoms (3di)								
Social communication deficits (cuttoff: 10)	—	3.84 (3.79)	13.26 (4.17)	<0.001	—	3.45 (3.70)	13.97 (4.02)	<0.001
Resticted and repetitive behaviors (cuttoff: 3)	—	1.18 (1.69)	5.85 (2.54)	<0.001	—	1.23 (1.80)	6.33 (2.30)	<0.001
ADHD symtoms (CPRS-R:S)								
Inattention (cuttoff: 9)	—	11.64 (4.27)	9.75 (4.62)	0.24	—	11.63 (4.32)	9.11 (4.73)	0.18
Hyperactivity (cuttoff: 7)	—	9.21 (4.42)	5.72 (3.41)	0.01	—	10.09 (4.66)	5 (3.46)	<0.005
ADHD index (cuttoff: 20)	—	23.79 (6.72)	18.72 (7.01)	0.04	—	24 (7.42)	17.43 (7.09)	0.03

*ANOVA two-tail test. IGT-C: Iowa Gambling Task for Children; PDG-C: Prisoner’s Dilemma Game for Children; 3di: Developmental, Diagnostic and Dimensional Interview; CPRS-R:S: Conner’s Parent Rating Scale Revised: Short form.
