# Supplementary material for: Neural markers of social and monetary rewards in children with Attention-Deficit/Hyperactivity Disorder and Autism Spectrum Disorder
Source: Sci Rep. 2016 Jul 28;6:30588. doi: 10.1038/srep30588 (PMC4964357; doi:10.1038/srep30588)
Supplement: Supplementary Information [file srep30588-s1.pdf]

Supplementary Information

Title: Neural markers of social and monetary rewards in children with Attention-Deficit/Hyperactivity Disorder and Autism Spectrum Disorder.

Author names: *Gonzalez-Gadea, Maria Luz; Sigman, Mariano; Rattazzi, Alexia; Lavin, Claudio; Rivera-Rei, Alvaro; Marino, Julian; Manes, Facundo; Ibanez, Agustin*[*]

1. Data exclusions

Some participants were excluded from the data analysis. For the IGT-C analysis two children were excluded because the data was incomplete (i.e., one child from the control group who did not complete the task) or excessively noisy (this was the case for one control ADHD children). Participants were also excluded from the PDG-C analysis because they failed to complete the task (one from the control group and three from the ASD group), yielded excessively noisy data (one from the ADHD group and two from the ASD group), or had less than 70% accuracy in responding to control question (one from the control group, two from the ADHD group, and one from the ASD group).

2. Influence of high ADHD symptoms in ASD sample

In order to further explore whether a subgroup of ASD children with high ADHD symptoms presented differential value-related brain processing, we performed two additional analyses: (1) a Monte Carlo permutation test to compare fERN modulations between conditions in two subgroups of children with ASD: those with high ADHD symptoms (above the clinical cutoff on the Conners' Parent Rating Scale Revised: Short

form) and, those with low ADHD symptoms (below this clinical cutoff). (2) correlations between the ADHD symptoms and the EEG measures in the ASD sample.

First, the Monte Carlo permutation analyses revealed that both ASD participants with high ADHD symptoms and ASD children with low ADHD symptoms exhibited greater fERN responses to losses than to wins in both types of choices in the IGT-C (DDs and high-loss frequency decks). In the PDG-C, both groups exhibited significant greater fERN responses to the cooperative options compared to the betrayal options (see Fig. S1). In sum, results showed that both groups presented similar fERN modulations than those observed in the entire ASD sample.

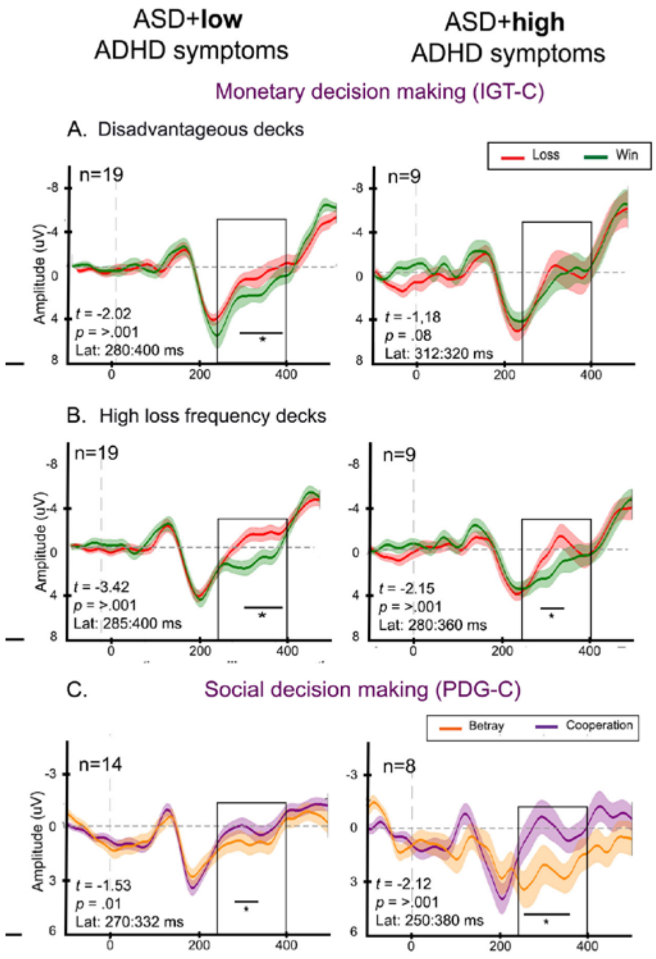

Supplementary Figure S1. FERN modulations in ASD individuals with high and low ADHD symptoms. ERPs of ASD participants with high ADHD symptoms (i.e. above the clinical cutoff of

the Conners' Parent Rating Scale Revised: Short form) and ASD children with low ADHD symptoms.**(A)** the win and loss conditions from the disadvantageous options of the IGT-C, **(B)** win and loss conditions from the options with high-loss frequency in the IGT-C, and **(C)** the cooperative and betrayal conditions in the PDG-C. The plots show grand-average ERP time courses at the Fz electrode in microvolts. Shaded bars around the ERPs indicate s.e.m. The thick, black horizontal lines indicate the temporal extents of the significant differences between the conditions. The black rectangular box indicates the fERN time window within which the conditions were compared. The number of subjects, latency of significant effects and *t* an *p* values are included in each plot.

In the second analysis, we performed non-parametric correlations between the ADHD symptoms and the EEG measures (fERN modulation and ACC activation, see details in section 6 of Supplementary Information) in the ASD sample. Supplementary Table S6 shows that ADHD symptoms were not significantly correlated with EEG measures in the ASD group.

Supplementary Table S6. Correlations between EEG measures and ADHD symptoms in the ASD group.

		Inattention symptoms		Hyperactivity symptoms		ADHD index	
		*r**	*p*	*r**	*p*	*r**	*p*
fERN modulation	IGT-C: Disadvantageous decks	.06	.82	-.20	.43	-.03	.92
	IGT-C: High-loss frequency decks	.21	.39	.15	.55	.09	.73
	PDG-C	-.29	.31	.08	.78	-.24	.41
ACC activation	IGT-C: Disadvantageous decks	.20	.43	-.31	.21	.09	.71
	IGT-C: High-loss frequency decks	.31	.21	-.29	.24	.09	.72
	PDG-C	-.20	.51	-.05	.87	-.26	.39

*Rho Spearman coeficient. IGT-C: Iowa Gambling Task for Children; PDG-C: Prisioner's Dilemma Game for Children.

3. MDM task: IGT for children (IGT-C)

Each trial began with the presentation of the two decks (see Fig. 1A). After the participant responded (between 300 and 900 ms), the stimulus was replaced with a fixation cross that was displayed for a random interval ranging between 500 and 800 ms. Next, the feedback display was presented for 500 ms. The winning feedback

consisted of a green card with the amount won depicted on top. The loss feedback consisted of a card split down the middle; the amount of the win in green on the top, and the amount of the loss was presented in red on the bottom. Finally, a black screen was displayed for an interval from 500 to 800 ms random to indicate the start of the next trial. Thus, the inter-trial intervals ranged from 1300 to 2700 ms.

The participants were instructed to select a card from either the left or the right deck by pressing 1 or 2, respectively. Their goal was to maximize an initial capital ($120) that was represented by a money bar that was displayed at the beginning of the task. After 20 choices, an outcome display revealed the money bar with an update of the total amount won or lost up to that point (cumulative feedback). The participants resumed the task by pressing the spacebar. The task finished after the 8th outcome display (160 trials).

Both versions contained an Advantageous Deck (AD) with small wins ($2) and a Disadvantageous Deck (DD) with high wins ($4). Every card from both decks included a win; consequently, the reward frequency was constant across the versions (100%). However, the magnitudes and frequencies of loss differed across the decks and versions. In version A, the loss frequency was low (40%) for the AD and high (60%) for the DD. In version B, the loss frequency was inverted between the decks, i.e., the loss frequency was high (60%) for the AD and low (40%) for the DD. In version A, the identification of the AD was facilitated by the low-loss frequency associated with this option. However, this identification was more difficult in version B due to the high-loss frequency of the AD (see Fig. 1B). For data analysis, the decks from the two versions of

the IGT-C were split into four conditions: ADs, DDs, low-loss frequency decks, and high-loss frequency decks.

The participants were blind to both the distributions of wins and losses between the decks and the numbers of trials in each version. The participants were informed that they would receive chocolates after task completion according to their accumulated money. Specifically, one chocolate was given for $120, two for profits between $120 and $180, and three for profits greater than $180. All participants performed both versions, which order was counterbalanced between subjects. In both versions, we counterbalanced the left and right localizations of the AD and DD. Prior to the initiation of the task, the participants performed a short training task (16 trials).

Behavioral measures: We counted the number of cards selected by each participant in each deck and version. A repeated measures ANOVA comparing these within-subjects variables (decks: AD and DD; versions: A and B) revealed no significant group differences (F (2, 60) = .94, p = .39). No interactions were observed between groups and decks (F (2, 60) = .62, p = .54) or between groups and versions (F (2, 60) = -2.34, p = .980). As expected, we found main effects of decks (F (1, 60) = 27.39, p < .001) and versions (F (1, 60) = 19.20, p < .001): participants selected more cards from the AD than the DD, and from version A than version B (see Supplementary Fig. S2). Thus, ADHD, ASD, and control children performed the IGT-C similarly, all groups preferring ADs over DDs.

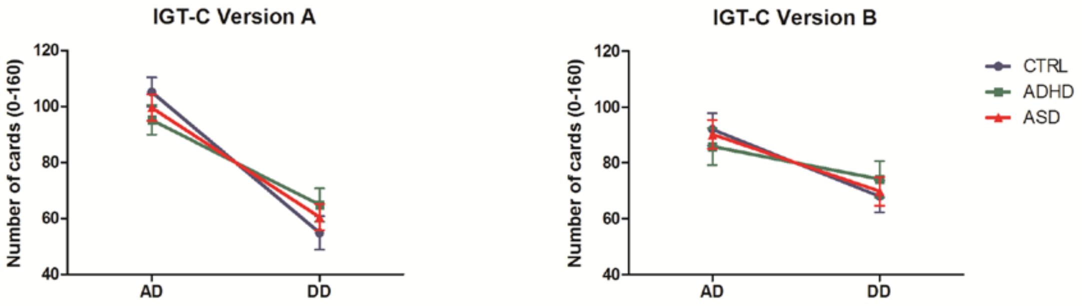

Supplementary Figure S2. IGT-C behavioral results. Mean and s.e.m of the number of cards selected from each deck and version of the task.

4. SDM: PDG for children (PDG-C)

In the PDG-C, the participants observed a virtual game between two players, i.e., Simon and Peter (see Fig. 2A). Simon was introduced as a nice and friendly boy, whereas Peter was presented as a naughty and rude boy. The participants were instructed that the players were in different rooms and independently selected whether to cooperate with each other or betray the other player in each trial. When both players cooperated, both won 3 points; when both players betrayed each other, both won 1 point; and when one player betrayed and the other cooperated, the first player won 5 points, and the player who cooperated did not win any points. The participants were instructed to pay attention to Simon's decisions because they would receive a reward according to the points that he accumulated. Each cooperation and betrayal condition includes two possible outcomes (see Fig. 2B): the cooperation condition includes when: (1) both players cooperated (20%) and (2) the fair player cooperated and the unfair player betrayed (52.5%), while the betrayal condition

includes when: (1) both players betrayed (15%) and (2) the fair player betrayed and the unfair player cooperated (12.5%).

Each trial began with a window depicting the decision of one of the players (200 ms) followed by a window that displayed the choice of the other player together with the first player's decision (900 ms, see Fig. 2C). The player's faces were shown in green when they cooperated and in red when they betrayed. The order in which each player decided first and the four possible outcomes were counterbalanced throughout the PDG-C. The selection of the fair player always appeared on the top of the screen, and the unfair player's choices were shown on the bottom. After both players' decisions, a black screen appeared for an interval that varied randomly between 50 and 100 ms. Next, the stimulus was replaced with a window that presented a question about who won or if the trial was a tie. This question was included to reinforce the participants' attentiveness to the task since they were informed that the lack of an answer would be punished with -1 point. The participants had 2000 ms to answer by pressing one of arrow keys on the lower row of the keyboard. When the participants answered incorrectly, a red cross appeared for 200 ms. Finally, a fixation cross appeared for 200 ms to indicate the start of the next trial. In cases of correct answers or the absence of an answer within 2000 ms, a fixation cross immediately appeared. Each trial lasted for approximately 1400 to 3500 ms.

After 40 choices, a display revealed an update of the total amount of points earned by each player up to that moment (cumulative feedback). The participants resumed the task by pressing the spacebar. The participants were informed that would receive chocolates according to the points accumulated by the fair player at the end of the

task. One chocolate was given if the fair player won between 200 and 400 points, two if he won between 400 and 600 points, and three for more than 600 points. The task was preceded by a short training session (8 trials), in which the control question had no time constraint. This allowed testing whether the participants understood the task, the consequences of fair decisions, and their own payoff. In case of incorrect answers, the practice session was repeated.

Behavioral measures: To compare responses to cooperation and betrayal options between groups, we calculated the number of correct answers to the control question (Who won?, see Fig. 2B, panel 4). First, for each participant, we calculated the proportion of correct responses in each condition (116 for cooperation options and 44 for betrayal options). A repeated measures ANOVA revealed no differences between groups (F (2, 55) = .85, p = .43) or options (F (1, 55) = .13, p = .71). Neither did these two variables interact (F (1, 55) = .20, p = .82) (see Supplementary Fig. S3). Hence, groups were paying equally accurate and attentive to task events.

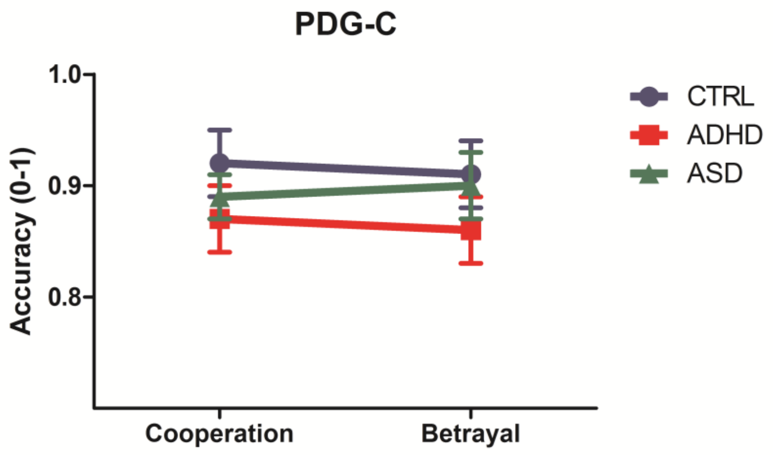

Supplementary Figure S3. PDG-C, behavioral results. Mean and s.e.m of the proportion of correct responses to the control question.

5. hdEEG data collection and preprocessing

Noisy channels and epochs were identified by calculating the normalized variance and then manually rejecting or retaining the data based on visual confirmation. The rejected channels were interpolated using spherical spline interpolations. There were no significant group differences in the percentage of epochs rejected in any of the experimental conditions (see details in Supplementary Table S1). The retained data were jointly re-referenced to the linked mastoid electrodes. These processing steps were implemented using custom MATLAB scripts that utilized EEGLAB functionality.

Supplementary Table S1. Mean and (s.e.m) of number of trials included in the EEG analysis and percentage of rejected trials in pre-processing.

		CTRL		ADHD		ASD		
		Number of trials	% Rejected trials	Number of trials	% Rejected trials	Number of trials	% Rejected trials	*p**
IGT-C	Disadvantageous decks	113.37 (9.38)	6.03 (1.68)	124.33 (10.56)	9.73 (1.96)	121.53 (8.02)	6.07 (1.44)	.21
	Advantageous decks	189.68 (10.32)	5.05 (1.48)	172.46 (11.62)	6.42 (1.73)	176 (8.82)	7.23 (1.27)	.52
	High-loss frequency decks	142.58 (5.08)	5.41 (1.43)	143.26 (5.71)	6.90 (1.67)	139.54 (4.34)	7.24 (1,23)	.61
	Low-loss frequency decks	160.47 (4.87)	5.42 (1.51)	153.53 (5.48)	7.96 (1.75)	158 (4.16)	6.77 (1.29)	.54
PDG-C	Cooperation options	106.84 (3.35)	7.89 (2.89)	99.82 (3.54)	13.94 (3.05)	103.50 (3.11)	10.77 (2.68)	.36
	Betrayal options	40.42 (1.27)	8.13 (2.89)	38 (1.34)	13.64 (3.05)	39.68 (1.18)	9.81 (2.68)	.41

*ANOVA was used for differences in the percentage of rejected trials in the EEG pre-processing. IGT-C: Iowa Gambling Task for Children; PDG-C: Prisioner's Dilemma Game for Children.

6. Influence of demographic variables in the EEG results

First, the main amplitude of the fERN component (250-400 ms window) was extracted for each subject and condition. Then, we calculated a composite score (called 'fERN modulation') by subtracting loss-minus-win in the IGT-C options and betrayal-minus-cooperation conditions in the PDG-C. Average results in the source measures (namely,

‘ACC activation’) were used to test group differences in the ACC ROI (see details in *Data Analysis* section). Next, we tested whether these EEG measures were influenced by age or gender. To test the influence of age in each group we performed correlations between EEG measures and age. No significant correlations were found in any group (see Supplementary Table S3).

Supplementary Table S3. Means (s.d) and group comparision of source amplitude at the right ACC *.

		CTRL	ASD	ADHD	CTRL/vs/ASD		CTRL/vs/ADHD		ASD/vs/ADHD	
					U	*p*	*U*	*p*	*U*	*p*
IGT-C	Disadvantageous decks	.091 (.206)	.104 (.288)	-.079 (.161)	253.5	.79	76	.02	107.00	.01
	High-loss frequency decks	.074 (.182)	.097 (.187)	-.037 (.117)	244	.63	82	.04	121	.02
PDG-C		.132 (.234)	-.087 (.247)	-.018 (.182)	101.50	.01	90	.04	137	.35

* Mean and SD values are expressed in 10^{-10}. Mann-Whitney test ($p < .05$) was used for group comparisons. IGT-C: Iowa Gambling Task for Children; PDG-C: Prisioner's Dilemma Game for Children.

Last, we explored gender differences in these EEG measures in the control and ADHD groups by using Mann-Whitney test (the ASD group was excluded from this analysis given the presence of only one girl). No differences in gender were found in the control and the ADHD group (see Supplementary Table S4). Together, these results suggest that age and gender were not significantly associated with the ERP and source activation results.

Supplementary Table S4. Means (s.d) and gender comparisions in the EEG measures in control and ADHD groups*.

		CTRL			ADHD		
		Boys (*N*= 12)	Girls (*N*= 8)	*p*	Boys (*N*= 11)	Girls (*N*= 5)	*p*
fERN modulation	IGT-C: Disadvantageous decks	.07 (.20)	.12 (.21)	.93	-.07 (.16)	-.10 (.18)	.72
	IGT-C: High-loss frequency decks	.14 (.19)	.04 (.13)	.19	-.01 (.11)	-.04 (.20)	.90
	PDG-C	.12 (.17)	.11 (.25)	.90	-.01 (.18)	-.07 (.18)	.53
ACC activation*	IGT-C: Disadvantageous decks	.02 (2.57)	.50 (3.57)	.88	.58 (2.15)	-.35 (2.31)	.69
	IGT-C: High-loss frequency decks	.95 (1.94)	1.34 (1,76)	.70	1.06 (1.67)	.95 (1.78)	.31
	PDG-C	1.50 (5.44)	.29 (4.26)	.80	-.70 (4.36)	2.64 (6,61)	.46

* Data and comparison for the ASD groups is not shown due to only one girl was included in this group. Mean and SD values for ACC activation measures are expressed in 10^{-10}. Mann-Whitney test ($p < .05$) was used for gender comparisons. IGT-C: Iowa Gambling Task for Children; PDG-C: Prisioner's Dilemma Game for Children.

Supplementary Table S2. Coordinates of the maximum peak of activation at the ACC ROI in each group.

		CTRL			ADHD			ASD		
		x	y	z	x	y	z	x	y	z
IGT-C	Disadvantageous decks	3.56	51.2	24.37	2.75	51.06	13.09	3.56	51.2	24.37
	High-loss frequency decks	4.66	50.31	23.66	8.81	46.55	25.4	8.81	46.55	25.4
PDG-C		10.19	49.31	22.45	10.19	49.31	22.45	4.66	50.31	23.66

IGT-C: Iowa Gambling Task for Children; PDG-C: Prisioner's Dilemma Game for Children.

Supplementary Table S3. Correlations between EEG measures and age in each group.

		CTRL		ADHD		ASD	
		*r**	*p*	*r**	*p*	*r**	*p*
fERN modulation	IGT-C: Disadvantageous decks	.15	.54	-.29	.31	-.07	.70
	IGT-C: High-loss frequency decks	-.20	.43	-.05	.86	.08	.66
	PDG-C	-.29	.23	.11	.67	.71	.76
ACC activation	IGT-C: Disadvantageous decks	.30	.23	.26	.37	.14	.48
	IGT-C: High-loss frequency decks	.63	.01	-.17	.57	.11	.57
	PDG-C	-.35	.14	.16	.55	.04	.86

*Pearson coeficient. IGT-C: Iowa Gambling Task for Children; PDG-C: Prisioner's Dilemma Game for Children.
